# Endothelial progenitor cells and burn injury – exploring the relationship

**DOI:** 10.1186/s41038-016-0028-x

**Published:** 2016-02-19

**Authors:** Derek A. Banyard, Blake O. Adnani, Satenik Melkumyan, Cheryl Ann Araniego, Alan D. Widgerow

**Affiliations:** Department of Plastic Surgery, Center for Tissue Engineering, University of California, Irvine, 200S Manchester Ave, Ste 650, Orange, CA 92868 USA

**Keywords:** Endothelial progenitor cells, Burn depth, Circulating angiogenic cells, Burn biomarker

## Abstract

Burn wounds result in varying degrees of soft tissue damage that are typically graded clinically. Recently a key participant in neovascularization, the endothelial progenitor cell, has been the subject of intense cardiovascular research to explore whether it can serve as a biomarker for vascular injury. In this review, we examine the identity of the endothelial progenitor cell as well as the evidence that support its role as a key responder after burn insult. While there is conflicting evidence with regards to the delta of endothelial progenitor cell mobilization and burn severity, it is clear that they play an important role in wound healing. Systematic and controlled studies are needed to clarify this relationship, and whether this population can serve as a biomarker for burn severity.

## Background

The nature and extent of burn injuries play important roles in determining the body’s pathophysiological response to this trauma. Approximately 450,000 people seek medical treatment for burns every year in the United States. Of these, approximately 40,000 are hospitalized, including 30,000 admissions to the 127 medical centers that specialize in burn care [1]. The pathophysiological response to thermal trauma is different when compared to other forms of injury. One particular element that is beginning to garner interest involves the systemic mobilization of endothelial progenitor cells (EPCs). Studies have shown that blood drawn from patients with burn wounds tend to have EPC counts which deviate significantly from typical basal concentrations [2–4]. Examining the correlation between human EPC levels and burn injury could indicate whether EPC counts are related to recovery time, potentially serving as an indirect parameter for injury severity as well as an indicator for survival [2–4].

The study of EPCs and their role in vasculogenesis has increased exponentially since Asahara et al. first described the isolation of cells from both human and mouse peripheral blood that contribute to endothelial cell (EC) formation and postnatal neovascularization [5]. Increasingly, evidence is mounting for the use of circulating EPCs as a biomarker for various diseases. For example, a recent study demonstrated that EPC numbers and function are significantly decreased in children diagnosed with systemic lupus erythematosus (SLE) when compared to patients with juvenile idiopathic arthritis and matched control subjects. Mohan et al. suspect the vascular damage observed in these patients is triggered by type I interferons, as is seen in patients with adult-onset SLE [6]. In the setting of oncogenesis, tumor development and metastasis is dependent on a sustained vascularization capability. As such, numerous studies have demonstrated that circulating EPCs directly correlate with tumor stage, size, microvessel density, and serum vascular endothelial growth factor (VEGF) concentration [7]. Extensive research has also shown that in the setting of ischemic brain injury, circulating EPC levels correlate with neurovascular remodeling and repair via paracrine mechanisms as well as direct differentiation [8]. Currently, the vast majority of EPC biomarker research is being conducted in cardiovascular medicine. Specifically, the conditions that comprise metabolic syndrome including coronary artery disease [9] and diabetes mellitus [10], have been consistently associated with fewer circulating EPCs [11].

The evidence with regards to EPC mobilization following trauma, and particularly thermal injury is less clear. Early studies have reported an immediate release of EPCs into the bloodstream of human patients after burn insult that is strongly correlated to the depth and extent of the burn [3, 12]. More recently, Zhang et al. used a systematic approach to induce burn wounds in mice and observed systemic mobilization of EPCs that negatively correlated with the severity of the burn both in terms of time and magnitude [13]. Subsequently, the same group at Johns Hopkins has conducted a number of in vitro studies implicating hypoxia-inducible factor 1α (HIF-1α) and its downstream mediators as critical to the deployment and recruitment of EPCs [14–16].

Regardless of their response, EPCs have a clear role in postnatal vasculogenesis and thus, the correlation between EPCs and burn injury depth represents a potential therapeutic target and/or diagnostic for the treatment of thermal injuries [17]. Burn injuries typically result in damage to the vasculature. Therefore, our group is interested in exploring the relationship between EPC mobilization/homing and vascular injury. Restoration (angiogenesis) or *de novo* formation (vasculogenesis) of the vascular network lost after burn trauma is necessary for the delivery of oxygen-rich blood containing the cellular response needed for prompt healing. However, before exploring this relationship, one must first look at the various characteristics used to define EPCs in the literature.

## Review

### Defining EPCs and circulating angiogenic cells

**Fig. 1:**
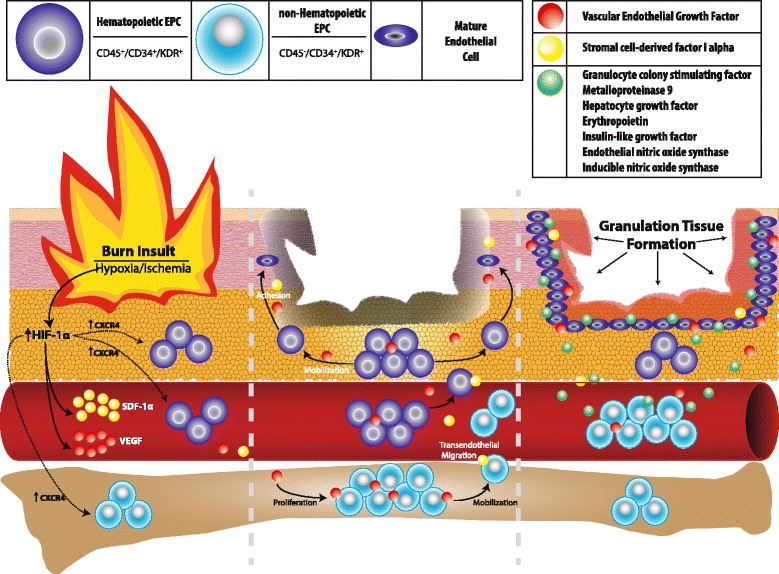
Proposed endothelial progenitor cell (EPC) involvement after burn injury. Burn insult and resulting hypoxia/ischemia lead to the upregulation of HIF-1α which promotes VEGF and SDF-1α secretion as well as increased CXCR4 expression on EPCs. VEGF is primarily responsible for the proliferation of hematopoietic EPCs (local tissue environment/blood stream) and non-hematopoietic EPCs (bone marrow) while SDF-1α promotes transendothelial migration into the bloodstream and soft tissue as well as adhesion at the site of tissue injury. Hematopoietic EPCs contribute to burn injury through direct attachment, maturation to endothelial cells and the formation of new vascular tissue, while non-hematopoietic indirectly aid in this formation through the secretion of various cytokines

In general, endothelial progenitor cells have been defined as circulating cells that express cell surface markers similar to those found on vascular endothelial cells, adhere to the endothelium at sites of hypoxia and ischemia and participate in new vessel formation [18]. The term ‘progenitor’ connotes that an EPC will eventually become a mature EC, enabling direct contribution to the formation of vascular tissue. Recent evidence, however, suggest that the term ‘endothelial progenitor cell’ may be a misnomer for this broadly defined population that exists in the bone marrow, circulation and in the local tissue microenvironment (Fig. 1).

Prior to 1997, postnatal angiogenesis was thought to be solely carried out by the migration of mature ECs while vasculogenesis occurred through angioblast-induction of hematopoietic stem cells (HSCs) [19]. This view changed when Asahara et al., utilizing a new method for the isolation of bone marrow-derived progenitor cells from human and mouse peripheral blood, demonstrated that these cells were capable of differentiating into endothelial cells and promoting neovascularization [5]. This group used magnetic beads to isolate cells that were positive for both CD34 and vascular endothelial growth factor 2 receptor (KDR), two markers that are highly expressed on activated ECs as well as HSCs [5]. While this marker combination became the standard for EPC selection by many throughout the field, this strategy was often criticized for its lack of CD45 investigation, whose presence indicates a hematopoietic identity [18].

Philosophically, a true ‘endothelial progenitor’ must include certain properties such as the ability to give rise to progeny displaying clonal proliferative potential, a differentiation capacity restricted to the endothelial lineage, and the ability to form lumenized capillary-like tubes in vitro. These cells must also possess the ability to form stable blood vessels in vivo that integrate into the host circulatory system once implanted [18]. The distinction, however, between the circulating cells that mature into ECs and those that indirectly contribute to neovascularization via paracrine mechanisms may not be necessary for the purposes of human clinical evaluation.

Early in vitro work on the peripheral blood mononuclear cells (MNCs) first described by Asahara et al. demonstrated that these cells (e.g., CD34^+^/CD133^+^/KDR^+^), though expressing various endothelial markers, also exhibit a number of myeloid/hematopoietic characteristics including the presence of CD45, CD14 and CD11b [11, 18, 20]. Despite this seemingly contradictory identity, these cells have been shown to exhibit proangiogenic activity such as participating in the restoration of blood flow in a murine ischemic hindlimb model, as well as improving cardiac outcomes in human patients after acute myocardial infarction [21]. Additional characteristics, such as the ability of these cells to bind acetylated low-density lipoprotein (acLDL) and lectin, have also been used to identify EPCs, but have proven to be nonspecific for a progeny that results in a true endothelial state [18]. In contrast, it has been proposed that only the CD34+/CD45- subset possesses the ability to progress to mature ECs [18]. To this point, Asahara et al. recently proposed that there are two subsets of endothelial progenitor cells: hematopoietic EPCs which are related to and derived from the same precursors as HSCs and nonhematopoietic EPCs, both of which contribute to postnatal neovascularization [22]. Thus, both can be classified as “circulating angiogenic cells” (CACs) when recovered from the bloodstream.

The pathway of EPC mobilization and homing is a complex one that is not fully understood. In the setting of burn injury, ischemia is the driving force behind EPC localization which is orchestrated by the upregulation of transcriptional factor HIF-1α [14–16]. Activation of this pathway leads to the expression of the key ligand stromal cell-derived factor 1α (SDF-1α) and its receptor CXC chemokine receptor 4 (CXCR4) [2, 11, 14–16, 23]. However, other factors strongly implicated in this process include VEGF, granulocyte colony stimulating factor, metalloproteinase 9, hepatocyte growth factor, and erythropoietin [24]. Once at the sight of injury, nonhematopoietic EPCs are thought to directly contribute to neovascularization by attachment and maturation into ECs, while hematopoietic EPCs contribute via paracrine signaling through the secretion of the cytokines such as VEGF, SDF-1α, hepatocyte growth factor, insulin-like growth factor, endothelial nitric oxide synthase and inducible nitric oxide synthase [22] (Fig. 1) .

### EPCs in relation to burn depth and severity (Table 1)

**Table 1:** Studies evaluating CACs in burn patients

Study	CAC characterization	Assessed correlation to burn depth severity?	Key findings
Gill et al., 2001 [12]	CD133^+^/KDR^+^/CD15^−^	No	Patients with > 15 % TBSA Burn → ↑VEGF systemically (at 6–12 h) which was followed by transient mobilization of CACs that peaked at 12 h and returned to baseline at 24–48 h.
Fox et al., 2008 [3]	CD45^dim/-^/CD133^+^/CD144^+^/KDR^+^	Yes	All pts with burns exhibited a significant rise in CACs that peaked at 24 h and returned to baseline after 72 h. There was a positive correlation between level of CACs and percent TBSA of burn. CAC levels also correlated strongly with VEGF & SDF-1α levels.
Piatkowski et al., 2009 [25]	CD34^+^/KDR^+^/acLDL^+^/lectin^+^	Yes	Significantly lower CAC count in pts with extensive burns (>25 % TBSA) than those with smaller burns (10–24 %) on admission. CAC levels rose in all burn pts, with extensive burn pt CAC levels not reaching a significant level until day 5 except in pts who did not survive.
Groger et al., 2010 [26]	CD34^+^/KDR^+^/acLDL^+^	No (animal model)	All animals sustained 30 % TBSA burn. Observed significant CAC drop during burn insult followed by rapid increase to ½ baseline levels at 2 h that remained stable for 48 h.
Pan et al., 2010 [27]	CD31^+^/KDR^+^/acLDL^+^	Yes	Ex vivo analysis of burn fluid demonstrated stronger paracrine signaling for MNC recruitment and CAC differentiation in DPTB fluid when compared to SPTB fluid.
Zhang et al., 2010 [13]	acLDL^+^/lectin^+^	Yes (animal model)	↑CACs in all burn groups, but day and magnitude of mobilization inversely proportional to degree of burn insult.
Foresta et al., 2011 [2]	CD45^+^/CD34^+^/CD133^+^/KDR^+^	No	Significant drop in CACs in burn pts on admission compared to control. Significant release in CACs both at day 1 and 12 not observed in control group.

Abbreviations: *CAC* circulating angiogenic cell, *KDR* kinase insert domain receptor (VEGFR-2), *acLDL* acetylated low-density lipoprotein, *TBSA* total body surface area, *MNC* mononuclear cell, *SPTB* superficial partial-thickness burn, *DPTB* deep partial-thickness burn, *VEGF* vascular endothelial growth factor, *SDF-1α* stromal derived factor 1α

In 2001, Gill et al. provided evidence that in humans, burn trauma results in a rapid release of VEGF that leads to a significant, but transient mobilization of CD133^+^/KDR^+^ cells, termed endothelial precursor cells, which usually returns to baseline after one to two days [12]. However, it was Fox et al. that first examined the relationship between EPC mobilization and burn severity. They collected blood from control patients as well as those who sustained superficial (*n* = 15) and full thickness (*n* = 4) burns at 24-hour time points for 3 days. The focus of their analysis was on nonhematopoietic EPCs based on a flow cytometry phenotyping protocol which quantified the CD45^dim/-^/CD133^+^/CD144^+^/KDR^+^ cells. This group observed a rapid and significant rise in systemic EPC levels that peaked within the first 24 h and correlated with burn depth severity. The statistically significant rise in EPC levels diminished after 48 h but was also strongly correlated with higher levels of VEGF and SDF-1α in the plasma that persisted for three days [3].

A group from Germany has also provided some clues regarding how MNC and EPC mobilization correlates with extent of burn injury. In their initial study, Piatkowski et al. collected blood samples at various time points during the first 5 days of hospitalization from 17 individuals who sustained burns ranging from 15 to 84 % total body surface area (TBSA) and compared it with 17 age-matched controls. They observed a significant rise in MNC counts on admission and at day 5, and these counts directly correlated with the extent of the TBSA of the burns. However, their analysis of EPC levels, which did not distinguish between hematopoietic and nonhematopoietic lineages (CD34^+^/KDR^+^/acLDL^+^/lectin^+^), demonstrated significantly lower EPC counts in burn patients when compared to the control group on admission. These levels did slowly rise, however, and reached statistical significance at day five except in those patients who did not survive. Notably, the most extensive burns (>25 % TBSA) were associated with significantly lower CAC levels when compared to those suffering less extensive burns [25].

This group went further when they described the first systematic porcine model for EPC mobilization after burn injury. Groger et al. quantified systemic MNC and EPC levels in pigs after subjecting them to 30 % TBSA full-thickness burns. They consistently observed a significant bimodal drop in MNC counts during the burn and at 12 h post-burn. The EPC count also dropped during the burn insult, but rapidly returned to baseline levels at 2 h and remained stable for the remainder of the 48 h post-burn observation period [26]. This study did not, however, examine whether EPC mobilization varies with the extent or depth of burn and is therefore limited in its ability to predict any correlations.

Using an innovative approach, Pan et al. closely examined burn blister fluid and resident human tissue from the sites of superficial (SPTBs) and deep partial thickness burns (DPTBs), classifications which serve as surrogate markers for burn severity. From a histological standpoint at day 7 post-burn, dermal tissue harvested from DPTBs demonstrated vast amounts of vascularity that was absent from the SPTB dermal tissue. In an in vitro transwell migration assay using burn blister fluids collected from the first 3 days post-burn, it was found that DPTB fluid induced significant mobilization of MNCs when compared to SPTB fluid and control media. And while burn blister fluid did not improve the KDR or von Willebrand factor (vWF) mRNA expression in ECs over control media, DPTB fluid was significantly superior to the other experimental conditions at promoting CAC differentiation from MNCs [27]. This study suggests that deeper burns are characterized by increased neovascularization over more superficial burns, and that factors present at the burn wound interface are likely involved in the recruitment and differentiation of CACs.

Similar to the group from Germany, Zhang et al. employed an animal model to systematically quantify CAC response to burn injury. These researchers analyzed the degree of murine CAC mobilization as it relates to burns of varying depth. They found an indirect correlation between burn depth severity and CAC mobilization as well as tissue perfusion, linking the two in the pathophysiological response to burn healing [13]. Thus, they postulate that deeper, more severe burns heal at delayed rates due to a lack of CACs, which in turn results in decreased angiogenesis and healing. Subsequently, this same group at Johns Hopkins sought to identify possible mechanisms underlying the delayed burn wound healing observed in elderly patients. In a mouse full-thickness burn wound model, they noted that older mice suffered delayed burn wound healing when compared to younger mice, and this was conferred by impaired mobilization of EPCs expressing CXCR4 as well as decreased systemic levels of levels of HIF-1α and SDF-1α [16]. This group went further by demonstrating that HIF-1α knockout mice suffer significant delays in burn wound healing that are associated with decreased mobilization of EPCs [15], and the addition of stabilized HIF-1α and EPCs led to rapid closure of these wounds in older mice compared to control [14].

Foresta et al. also examined the mobilization of CACs in patients suffering burns ranging from 15 to 57 % TBSA. While the study did not compare CAC mobilization to extent of burn insult, this group reported data up to 30 days post-injury. The hematopoietic EPC population (CD45^+^/CD34^+^/CD133^+^/KDR^+^) was quantified both on admission and at various time points during the study. Interestingly, they observed a bimodal distribution of CAC mobilization. The first peak was observed on day 1, similar to the findings of Fox et al., while the second and peak of greatest magnitude was seen on day 12, which they believe was associated with the escharectomy that all patients received on day 5 or 6. Similar to most other studies reviewed, this group observed a significant drop in admission CAC counts in the burn patients when compared to control.

### Future considerations: standardization of clinical assays and patient selection

The biggest impediment to conducting effective research on the role of CACs as a biomarker for burn severity is the lack of consensus with regards to phenotypic and physiological definitions. Despite this, one would find it difficult to dispute the role of the EPCs in neovascularization. For instance, Rignault-Clerc et al. isolated peripheral blood MNCs from individuals who sustained > 10 % TBSA burns within the first 24 h of hospitalization. This group used special culture techniques to obtain late outgrowth EPCs from both control and burn patients. These nonhematopoietic CACs are associated with the ability to directly contribute to vessel formation through their differentiation into mature ECs while lacking paracrine function. They found that late outgrowth EPCs from burn patients demonstrated significantly higher levels of VEGF secretion. However, this VEGF advantage did not necessarily translate into a clinical benefit as injection of these EPCs from both burn and control patients were equally effective at improving perfusion recovery in a murine model of hind limb ischemia [4].

Various methods were used to identify CACs in the studies reviewed. Most commonly, KDR was used in combination with either CD34 or CD133 to positively identify CACs [2–4, 12, 25, 26]. While either combination stain is sufficient for the identification of CACs, KDR^+^/CD34^+^ cells will be detected at ~200 times greater frequency than KDR^+^/CD133^+^, as CD133 denotes a more primitive progenitor phenotype [19]. And while other studies used acLDL and lectin positivity to identify CACs, this methodology is less practical for clinical translation as these assays require a cell culture step. KDR and CD34 are cell surface markers that can be easily quantified after isolating peripheral venous blood MNCs and analyzing with a flow cytometer. Lastly, we believe that CD45 should be excluded from the analysis of CACs. While this marker allows for the identification of hematopoietic versus nonhematopoietic CACs, we feel this distinction is unnecessary as both population of cells contribute to neovascularization [22] (Fig. 1) .

In terms of mobilization and recruitment, it appears that HIF-1α, VEGF and SDF-1α are most important for CACs [2, 3, 14–16]. It has been suggested that VEGF is more responsible for EPC proliferation, mobilization, adhesion and incorporation into damaged vessels while SDF-1α is primarily attributed to the facilitation of transendothelial migration and the induction of specific adhesion molecules [2]. Both of these cytokines are regulated by HIF-1α, which has clearly been demonstrated to show a significant role in burn wound healing in vitro [14–16]. Recently, angiogenin has been demonstrated to be a key paracrine signaler in superficial and deep partial-thickness burns for the promotion of neovascularization. Pan et al. demonstrated that angiogenin alone was able to induce CAC differentiation into ECs in the absence of VEGF-A, and that its absence was associated with a significant reduction in EC proliferation and new blood vessel formation in vivo [28].

One stark limitation in studying CAC mobilization as a biomarker for burn injury is the variability of patient co-morbidities and temporal relationship of the burn insult to the presentation to a tertiary facility for analysis. As evidenced by the first systematic animal-controlled study that measured blood levels during burn insult, thermal injury is associated with an immediate decline in CAC level [26]. This is a finding that was recently buttressed by Maluegha et al. who showed that mice subjected to electrical burns experienced an increase in VEGF and nitric oxide along with a simultaneous decrease in EPC counts [29]. From a clinical perspective, Foresta et al. and Piatkowski et al. observed declines in CACs of human patients on admission after burn insult [2, 25], however, where Piatkowski et al. didn’t observe a significant increase in CACs versus control throughout their study [25], Foresta et al. eventually observed a significant rise in CAC counts [2].

Whether there is a delay or immediate release, a number of studies report the eventual and significant mobilization of CACs in response to burn injury [2, 3, 12, 13]. And while some studies report this deployment as an isolated and transient event limited to the first 24–48 hours [3, 12], there is evidence that a secondary mobilization can occur as late as 12 days post-burn insult [2].

Clinically, elderly patients exhibit retarded wound healing after burns. The group at Johns Hopkins demonstrated that this mechanism of delayed and suboptimal healing is primarily mediated by the HIF-1α pathway and poorly functioning EPCs [14–16]. Based on the lack of age stratification and extreme variability in burn severity of the clinical studies presented here, there is reason to believe that there are various factors effecting CAC mobilization that have yet to be elucidated. In light of these complex and varied findings, it is clear that more research is needed in this arena.

## Conclusions

Endothelial progenitor cells comprise various cell populations that are CD34^+^/KDR^+^ and exist in the bone marrow, local tissue microenvironment and circulation. As has been evidenced in the cardiovascular literature, systemic levels of these cells likely reflect local tissue injury, especially in the setting of thermal trauma. While evidence is conflicting at this point, the use of CACs as a biomarker of degree of burn insult is promising. Further controlled and highly-powered studies are required to elucidate this relationship.

## Abbreviations

acLDL: acetylated low-density lipoprotein
CAC: circulating angiogenic cell
DPTB: deep partial-thickness burn
EC: endothelial cell
HSC: hematopoietic stem cell
KDR: kinase insert domain receptor (VEGFR-2)
MNC: mononuclear cell
SDF-1α: stromal derived factor 1 alpha
SPTB: superficial partial-thickness burn
TBSA: total body surface area
VEGF: vascular endothelial growth factor
HIF-1α: Hypoxia inducible factor 1 alpha
